# Tumor innervation and mitochondrial transfer in the cancer pathogenesis: perspectives on genitourinary malignancies

**DOI:** 10.3389/fonc.2025.1710038

**Published:** 2026-01-14

**Authors:** Lucas Assoni, Juliana Baboghlian, Roger Frigério Castilho, Leonardo Oliveira Reis

**Affiliations:** 1 UroScience, School of Medical Sciences, University of Campinas (UNICAMP), Campinas, São Paulo, Brazil; 2 Department of Pathology, School of Medical Sciences, University of Campinas (UNICAMP), Campinas, São Paulo, Brazil; 3 ImmunOncology, Pontifical Catholic University of Campinas (PUC-Campinas), Campinas, São Paulo, Brazil; 4 Institutos Nacionais de Ciência, Tecnologia e Inovação (INCT) UroGen, National Institute of Science, Technology and Innovation in Genitourinary Cancer, Campinas, São Paulo, Brazil

**Keywords:** tumor innervation, cancer immunology, mitochondria transfer, plasticity, oncogenesis, genitourinary cancer

## Abstract

Recent advances have significantly expanded our understanding of the roles played by mitochondria and tumor innervation in tumorigenesis. Once viewed primarily as contributors to energy production and metastatic dissemination, mitochondria are now recognized as central players in broader processes, including immune modulation within the tumor microenvironment, therapy resistance, and metastatic progression. Interestingly, the findings have eventually converged, and evidence now shows that mitochondria can be transferred from neurons to tumor cells, resulting in enhanced invasiveness. While these discoveries are promising, they also present new challenges that must be addressed. As the interconnection between neuroscience, oncology, and immunology continues to deepen, these insights open new avenues for the development of innovative therapeutic strategies. This review explores the most recent findings regarding nerve-cancer interaction, with a specific focus on genitourinary cancers, highlights their emerging intersections, and discusses how these insights may inform the development of novel therapeutic targets for cancer treatment.

## 1 Introduction

Cancer remains one of the leading causes of death worldwide, with the highest incidence of primary tumors observed in the breast, prostate, lung, and colorectal tissues. Despite advances in therapeutic strategies, projection models indicate an increase in both incidence and, particularly, mortality rates in underdeveloped countries when compared to developed ones, suggesting a trend to a significantly higher burden in countries with less developed healthcare systems (1). In the ongoing search for a better understanding of the processes that mediate cancer development, recent findings have highlighted the significant role of tumor innervation at various stages of tumor progression. Evidence suggests that neural involvement can either promote or suppress tumor growth, influencing the prognosis of the disease (2, 3). However, the mechanisms underlying cancer-induced innervation and the reprogramming of neural pathways remain unclear.

In a recent breakthrough work by Hoover and colleagues (4), it was demonstrated that in a breast cancer model, tumor cells can acquire mitochondria from cancer-associated neurons, and this acquisition correlates positively with increased metastatic potential. These findings suggest the need to explore additional factors previously overlooked, such as the interplay between cellular senescence, neuron-derived mitochondria, and outcomes.

This review discusses the most recent findings on the nerve-cancer intersection field, with a specific focus and perspectives on genitourinary cancers. It encompasses the following key themes: (i) a comprehensive understanding of the mechanisms underlying tumor innervation; (ii) deeper insights into the potential effects of mitochondrial acquisition by tumor cells from adjacent stromal cells, particularly neurons; (iii) elucidation of how these processes intersect in conferring energetic advantages, promoting immunomodulation, and enhancing tumor invasiveness; and (iv) the development of therapeutic strategies, either as standalone treatments or in combination, that effectively target these interconnected pathways.

## 2 Cancer neurogenesis: role and importance for tumor development

Recent studies have thoroughly discussed the importance of tumor innervation, which begins with the secretion of signaling molecules or extracellular vesicles (exosomes) that recruit and guide nearby nerves into the tumor microenvironment (TME) (3, 5). The TME is a highly complex and dynamic system, composed of diverse cellular elements embedded within an altered and vascularized extracellular matrix, in which the infiltration of nerve fibers, alongside cancer, stromal, and immune cells, plays a critical role in orchestrating tumor development through both direct and indirect mechanisms (6).

In addition to neurotrophic factors such as nerve growth factor (NGF), tumors secrete proteins, lipids, DNA, and RNA via exosomes, facilitating intercellular communication. Molecules carried in these exosomes, together with neurotransmitters, can modulate key biological processes in the TME, including angiogenesis, lymphangiogenesis, inflammation, and oncogene activation (7, 8). Some exosomal contents, such as small interfering RNAs (siRNAs), have been shown to inhibit neuronal outgrowth, while microRNAs like miR-34a regulate neural stem cell differentiation and neurogenesis.

Notably, tumor cells with suppressed exosome release exhibit reduced axonogenic potential compared to non-inhibited cells. In models of head and neck squamous cell carcinoma, for example, tumor-derived exosomes were found to promote neurite outgrowth and enhance tumor innervation (8, 9). Among the pro-neurogenic molecules found in exosomes are semaphorins, which can promote axonogenesis. Semaphorin 4F (S4F) is highly expressed in human prostate cancer cells (DU-145) and has been shown to induce neurite sprouting and increase neurite length by nearly threefold. Experimental studies have used S4F-targeting RNA interference to attenuate these effects, further supporting its role in tumor-associated neurogenesis (8, 10).

As neoplasia progresses, in general, tumor nerve density can increase to nearly twice that observed in non-neoplastic control tissue (8). Metastases and the degree of morbidity and consequently poor prognoses are often associated with high density of nerve cells, as in pancreatic ductal adenocarcinoma (11), oral cavity cancer (12) and perihilar cholangiocarcinoma (13). In prostate cancer, nerve density can be variable and diffusely located, found in greater quantity in marginal regions of the neoplasm, compared to samples of benign hyperplasia or normal prostatic tissue (14, 15). An immunohistochemical study using neural markers PGP9.5 and S100 located the highest nerve concentration in the tumor periphery, suggesting it is the result of expansive growth (15).

The newly formed neural networks within the TME can then regulate tumor function and promote a more aggressive phenotype, often associated with poorer prognosis, increased metastasis, and greater invasiveness. Among the underlying mechanisms, one key factor is the modulation of cellular behavior, which leads to both local changes, such as angiogenesis and tumor growth, and systemic effects, as altered immune responses and further progression of innervation (16). In addition to the local TME modulation, perineural invasion by tumors represents a huge issue as it relates to metastasis, often associated with worse prognosis, in a tumor-specific manner. The invasion mechanisms deployed by the tumor cells appear to imitate the interplay among the cells involved in the physiological neuronal tissue repair (17).

An interesting aspect of tumor innervation is the distinct roles that different types of nerves play across various tissues. Sympathetic nervous system (SNS) innervation is often associated with promoting tumor cell proliferation. In contrast, the parasympathetic nervous system (PSNS) and vagal innervation exhibit tissue-dependent effects, e.g., PSNS innervation correlates with tumor progression in prostate cancers (PC), but it has also been reported to possess an antitumorigenic role in pancreatic cancer (2, 3). Similarly, sensory nerves contribute to cancer development, predominantly acting as tumorigenic factors across a wide range of tissues (2, 3).

The presence of neural cells also influences the immune response within the TME. In cases where innervation exhibits tumorigenic properties, immune suppression is often observed. Consequently, both the type and density of nerve fibers are considered prognostic indicators of disease progression and predictors of tumor spread, morbidity, and mortality (18). Nerves interact with the immune system by detecting pathogen-associated molecular patterns (PAMPs), damage-associated molecular patterns (DAMPs), and immune mediators (19). Additionally, neurotransmitters can modulate the immune response. For instance, norepinephrine has been shown to exert immunosuppressive effects by impairing dendritic cell function and reducing the migratory capacity of neutrophils and T cells, as well as diminishing natural killer (NK) cytotoxic activity (20). Neurotransmitters associated with sensory nerves also possess a role in immune regulation, as calcitonin gene-related peptide (CGRP) can alter cytokine expression and T-cell recruitment and distribution (including CD4^+^, CD8^+^, and regulatory T cells). Meanwhile, substance P triggers the release of pro-inflammatory cytokines and exhibits an anti-tumor function. As with other aspects of innervation, these effects are tumor-type specific and may be either pro- or anti-tumorigenic (18, 19).

The oncogenic role of PSNS innervation could be related to the anti-inflammatory role of acetylcholine and its receptors, which are generally associated with changes in pro-inflammatory cytokine levels such as IL-1β and TNF-α. Cholinergic activity was also associated with NK and CD8^+^ T-cell migration. On the other hand, activation of adrenergic receptors by the SNS promotes the expression of immunosuppressive factors such as arginase-1, PD-L1, and myeloid-derived suppressor cells. Despite the immunosuppressive roles, anti-tumorigenic effects of the SNS have also been documented (18).

Researchers have also investigated the correlation between carcinogenesis and stress, identifying interrelationships with depression and social and lifestyle stress factors. These factors are associated with poorer outcomes in individuals experiencing emotional burdens, particularly those who develop cancer at some point in their lives (2, 21). Among the potential explanations, sympathetic nerve activation by psychological triggers might act as a cancer-promoting element (2).

## 3 Metabolic needs and advantages of cancer neurogenesis during growth and metastasis

In the “cancer swamp” model proposed by Amend and Pienta (22), the authors draw an analogy between the microenvironment changes observed within the TME to those observed during the eutrophication of an ecological environment. This transformation results in the collapse of the host’s natural equilibrium, culminating in what may be termed “ecosystem collapse”, manifesting, as organ failure and death in oncological terms. When considering the needs for tumor growth, maintenance, and dissemination, many of the changes observed are associated to support the metabolic demands. However, the environment is shared between the malignant cells and components of the immune system responsible for their elimination, in a competitive dynamic. In this conjecture, nutrient availability and energy abundance emerge as critical determinants for success, acting as evasion mechanisms against immune cells which lacks the commensurate means to compete in this setting (23, 24).

One major characteristic of the TME are the changes in the preferred metabolic pathways. Since the environment is often deprived of oxygen, the cancer cells shift their metabolism from the typical and more effective glucose oxidation to the less-effective process of fermentation, in a process called the Warburg effect. This metabolic reprogramming leads to reduced availability of nutrients and accumulation of lactic acid, resulting in acidification of the microenvironment (22). However, this phenomenon is not yet fully understood. A different viewpoint proposed by Wang and Patti suggests that the Warburg effect is a product of a mitochondrial overload (22). Since cancer cells generally have functional mitochondria (25, 26), during cell proliferation, the glycolysis rate elevates to a point that the subsequent steps of the glucose oxidation are overridden, as a consequence of the mitochondrial saturation (27).

Another major metabolic shift observed in genitourinary cancers involves lipid-associated pathways. In prostate cancer, reprogramming of lipid metabolism is directly linked to more aggressive tumor phenotypes that progress to resistance to treatment (28). Transcriptomic analyses have shown that patients with subtypes characterized by increased lipogenesis-associated metabolic pathways exhibit higher Gleason scores, poorer prognosis, and reduced survival rates. These tumors are also associated with an immunologically suppressed TME, with increased PD-L1 expression and the presence of M2 macrophages and regulatory T cells (Tregs). Conversely, this same group displayed downregulation of HLA genes and reduced infiltration of CD8^+^ and CD4^+^ T cells (29). Among the key regulators of this metabolic reprogramming, sterol regulatory element-binding proteins (SREBPs) mediate lipogenesis and cholesterogenesis, while also modulating androgen receptor expression, ROS production, and lipid accumulation in cancer cells. Elevated SREBP expression correlates with higher Gleason scores and aggressiveness in prostate cancer, contributing to castration resistance. Conversely, inhibition of SREBP activity using fatostatin exerts antitumoral effects, which are further amplified when combined with docetaxel (30–32). Fatty acid synthase (FASN) overexpression is another tumoral marker, particularly in castration-resistant subtypes, and has been positively correlated with lethality (33). FASN suppression inhibits tumor invasiveness and proliferation and increases sensitivity to enzalutamide (33, 34). In breast cancer cells, when inhibiting FASN, mitochondrial-induced apoptosis is induced and the cell is marked for elimination (35, 36). Fatty acid–binding proteins (FABPs) are also key contributors to lipid dysregulation in prostate cancer, as the upregulation of certain FABP family members correlates with higher Gleason scores, increased invasiveness, and poorer clinical outcomes (37, 38).

Cholesterol and its fractions also play a role in prostate cancer, as elevated intracellular cholesterol levels have been associated with tumor growth, resistance to androgen deprivation therapy (39, 40) and macrophage polarization to the M2 phenotype (41). Low-density lipoprotein (LDL) cholesterol promotes cancer stemness, particularly in the presence of 5α-dihydrotestosterone (DHT), leading to lipid accumulation in tumor cells and linking obesity, androgens, and prostate cancer. The same study also established the link between obesity, androgens and prostate cancer (42). Conversely, cholesterol efflux mediated by high-density lipoprotein (HDL) exerts antiproliferative effects, independent of androgen receptor status (43). Nonetheless, associations between cholesterol (and its fractions) with prostate cancer risk remain inconsistent. In the REDUCE trial, higher HDL levels and statin use correlated with reduced chronic prostatic inflammation, when compared to the group with high LDL levels (44). However, when evaluating patients that did not use statins and had high PSA values, only HDL was associated with higher cancer risk (45). Nevertheless, other populational studies associated higher cholesterol levels, whether or not in the presence of statins, with potential higher risks of prostate cancer (46–48).

Similar metabolic alterations are observed in bladder cancer, where increased lipid content within tumor cells is associated with more aggressive phenotypes and resistance to cisplatin, immune checkpoint blockade therapy and radiotherapy (49–52). Additionally, urothelial bladder cancer cells resistant to cisplatin also display enhanced lipid metabolism (53). Neuronal-surface-associated glycans are also expressed in bladder cancer cells; notably, GD2 ganglioside expression in muscle-invasive cell lines correlates with elevated intracellular fatty acid levels (54). Just like in prostate cancer, SREBP-regulated pathways are implicated in tumorigenesis and worse clinicopathological outcomes (55). Likewise, FASN expression may promote bladder cancer progression, immune suppression, and resistance to gemcitabine and cisplatin, as FASN inhibition has been shown to counteract these effects (56, 57). Additional pathways and regulators may also contribute to the lipid metabolism reprogramming observed in bladder cancer (58–61) and urothelial carcinoma (62, 63). Epidemiological evidence further indicates that elevated serum triglyceride levels are associated with an increased risk of bladder cancer (64, 65).

Renal cancers also exhibit marked alterations in lipid metabolism. In clear cell renal cell carcinoma, transcriptomic analyses reveal upregulation of lipid metabolic and inflammation-related pathways correlated with reduced patient survival expectancy (66). Inhibition of lipid accumulation, in turn, reduces tumor stemness (67). The metabolic shift also suppress the activity T-cells found within the TME, as they are less able to infiltrate and exert its functions (66). Among the main regulators and pathways involved in the intracellular lipogenesis process, and its subsequent pro-tumorigenic effects, we can point to SREBPs (68, 69), FASN (70–72), FABPs (73–75), AUP1 (76) and the hydroxyacyl-CoA dehydrogenase alpha subunit (HADHA) (77, 78).

An unfavorable lipid profile, namely, low HDL and elevated LDL, triglyceride, and cholesterol levels, may also represent a risk factor in clear cell renal cell carcinoma (67, 79). Moreover, controlled trials indicate that interventions leading to improved lipid profiles are associated with better overall prognosis (80, 81). However, as in prostate cancer, findings remain inconsistent, with some studies reporting that higher cholesterol and triglyceride levels correlate with improved outcomes (82, 83).

Overall, while a shift in lipid metabolism is a hallmark of genitourinary cancers, further research is required to actually correlate the pre-clinical findings to the clinical observations. Nevertheless, the critical role of mitochondria in cancer initiation and subsequent metastasis is increasingly evident, while also expanding from the metabolic-only aspect to a more systemic approach. In the model proposed by Hoover and colleagues, the stemness potential of the cancer cells is significantly enhanced through the acquisition of mitochondria from neurons, as the organelles were found at an increased number in the metastatic sites when compared to their primary site. According to their hypothesis, the increase in energetic potential confers a substantial adaptive advantage, thereby promoting metastatic success (4).

Mitochondrial transfer was also observed from CD8^+^ T-cells to cancer cells. When tracking the mitochondria from isolated T-cells, it was observed via an RNA sequencing-based model that murine lung cancer cells were able to acquire the organelles in an *in vitro* co-culture model. By applying the same RNA sequencing method in patients’ samples with basal cell or esophageal squamous cell carcinomas, an increase in the expression of genes associated with energy production, cytoskeleton modelling, and TNF-associated pathways was shown. Overall, the correlation between cell proliferation and mitochondrial acquisition was connected to a phenotype with a worse clinical prognosis (84). Recent studies have reported mitochondrial transfer as a contributor to cancer progression in various tumor types. In breast cancer, this transfer has been observed from cancer-associated fibroblasts (85) and adipose stem cells (86); in colon cancer, from adjacent epithelial cells (87) and bone marrow-derived mesenchymal stem cells (88); in glioblastoma, from astrocytes (89); and in ovarian and gastric tumors, from carcinoma-associated mesenchymal stromal cells (90, 91). Interestingly, in bone cancers, mitochondrial transfer from osteocytes may serve as an antitumor mechanism (92). Collectively, these findings highlight mitochondrial transfer as an emerging mechanism of resistance against host defense strategies.

In non-neoplastic lesions, the preservation of mitochondria is also of great importance in tissues. Mitochondrial lesions caused by acute renal failure cause mitochondrial fission through the signaling mechanism exerted by the DRP1 protein, leading to cellular apoptosis. It has been demonstrated that the inhibition of DRP1 by miRNAs from extracellular vesicles of renal stromal mesenchymal cells was able to inhibit mitochondrial fission, representing a promising treatment to contain damage in renal lesions (93). Similarly, the anti-apoptotic mechanism caused by the transfer of mitochondria from bone marrow-derived mesenchymal stem cells prevented death and improved recovery of cardiomyocytes in a model of blood ischemia and reperfusion injury (94). Interestingly, when mitochondria from a healthy cell enter a damaged or stressed cell, mitochondrial transfer may function as a recovery mechanism for cells that have lost mitochondrial functionality. In a co-culture model of pheochromocytoma using PC12 cells, it was demonstrated that mitochondrial transfer from healthy PC12 cells to UV-stressed PC12 can promote the recovery of these cells, preventing the early stages of apoptosis, prior to caspase-3 activation (95).

Immune responses are also influenced by mitochondrial activity through the release of damage-associated molecular patterns (DAMPs). Among the newly reported regulatory mechanisms is the fumarate hydratase (FH), an enzyme of the tricarboxylic acid (TCA) cycle. Dysfunction of TCA cycle is closely associated with cancer establishment and its progression, and one of the mechanisms behind it is the loss of FH activity, leading to intracellular fumarate accumulation. Mutations in the fumarate hydratase gene that cause FH loss of activity are associated with rare types of kidney cancer (96), which may become more aggressive (97). However, it has also been shown that fumarate functions as a signaling molecule for innate responses. In a renal carcinoma mouse model lacking FH-encoding alleles, the release of mtDNA in mitochondrial-derived vesicles triggered an IL-6-mediated inflammatory response (98). In other *in vitro* and *in vivo* FH inhibition models, the intervention led to the suppression of interleukins IL-1β, IL-1, and IL-10, while also increasing TNF levels. In addition to the cytokine profile modulation, FH also plays a role in macrophage activation (99). Other TCA cycle-derived metabolites, such as succinate and itaconate, also contribute to immune regulation. Succinate acts as a pro-inflammatory molecule by inducing IL-1β production, whereas itaconate has anti-inflammatory effects by reducing reactive oxygen species (ROS) production and inhibiting inflammatory pathways (96). Another regulation mechanism mediated via the release of metabolites is the modulation of the T-cell response. One example is the secretion of lactate, which can lead to inhibition of CD8^+^ T-cells as well as activating regulatory T-cells, while also polarizing macrophages to an M2 phenotype and repressing IFNγ secretion (24). While more data is needed, mitochondrial metabolites produced during metabolic processes may offer insights into therapeutic targets or pathways involved in cancer development.

Whether functioning as part of the cellular ATP supply or exerting influence in its surrounding microenvironment, mounting evidence supports the central role of mitochondria in tumorigenesis. Although the full range of benefits conferred by the acquisition of neuron-derived mitochondria remains to be elucidated - whether these benefits are limited to enhanced energy production or also involve roles in immune regulation, evasion, and cellular reprogramming - there is an interesting prospect in exploring mitochondrial function from a more systemic perspective.

## 4 Mitochondria and cell senescence in a neurogenesis context

Cellular senescence is implicated in both the natural aging process and in cases of cancer development. Characterized by the accumulation of cellular damage leading to a permanent loss of proliferative self-repair capacity, senescent cells are typically cleared by immune cells during tissue repair. As the individual ages, this process becomes flawed, allowing senescent cells to persist and potentially develop oncogenes (100). A series of landmark articles by Hanahan and Weinberg has outlined the biological capabilities that enable cancer development, termed the “hallmarks of cancer”. In their most recent report, senescent cells, which were originally viewed as tumor suppressive, have been proposed as an emerging hallmark of cancer. This shift stems from several factors, including their increased likelihood of harboring DNA mutations. Senescent cells can actively support neoplastic progression through the secretion of bioactive molecules known as the senescence-associated secretory phenotype (SASP). These factors stimulate neighboring malignant cells and reshape the tumor microenvironment by promoting angiogenesis, enhancing cell invasion through protease release, driving proliferation, suppressing antitumor immunity, and activating anti-apoptotic pathways. Evidence also suggests that cells undergoing transient senescent states, as well as stromal cells present within the TME that have undergone induced senescence, exhibit pro-oncogenic behavior (101).

Mitochondria in senescent cells exhibit significant alterations compared to those in non-senescent cells, including changes in spatial organization, number, physiological characteristics, and overall metabolic functionality. These changes are marked by reduced oxidative phosphorylation, potentially favoring the glycolytic process. Furthermore, the release of DAMPs, reactive oxygen species (ROS), and other metabolites, such as fumarate, also promotes the senescent phenotype in neighboring cells. These mitochondrial modifications may also contribute to resistance against apoptosis triggered by both intrinsic and extrinsic signaling pathways (102). In addition to the previously discussed context of renal cancers and fumarate accumulation, this process can also interfere with cellular senescence through epigenetic modifications, thereby enabling senescence bypass and promoting a malignant phenotype (103).

Studies examining mitochondrial transfer between senescent and proliferating cells have shown that, although both cell types can serve as donors and recipients, senescent cells predominantly function as mitochondrial donors (104). However, this raises an important question: could this mechanism also rescue cancer cells and hinder its elimination following therapeutic intervention or immune surveillance of the host? In studies using the MCF7 breast cancer cell line, the spontaneous acquisition of mitochondria from endothelial cells was shown to increase resistance to doxorubicin, a chemotherapeutic agent whose mechanism of action includes the induction of apoptosis through mitochondrial damage (105).

Arguably, the biggest novelty regarding the neural-tumoral axis is the transfer of mitochondria from neural to cancer cells (4), however, it remains to be fully elucidated whether the transfer of mitochondria from adjacent neural cells found in the tumoral stroma has similar mechanisms as the ones described above, warranting further investigation, due its enhancement of the metastatic potential. In this context, cancer-induced tumor innervation, coupled with its potential for mitochondrial transfer, may offer a novel perspective for therapeutic intervention, since the full extent of the functional and therapeutic benefits conferred by mitochondrial transfer to tumoral cells within the TME is yet to be completely understood.

## 5 Mitochondria and innervation as targets in cancer therapy

While recent studies have already explored the concept of targeting the mitochondria of a tumor cell in therapies, the idea of the cancer-induced innervation associated with mitochondrial transfer from either novel axons or senescent cells present within the TME sheds new light on this issue. The idea of precision medicine targeting mitochondrial metabolism is promising, as it is challenging, since toxicity, specificity, and cell reprogramming are huge bottlenecks for whichever therapy proposal. As there is evidence pointing to a mitochondrial role for PD-1 immunotherapy resistance, multifaceted approaches involving mitochondrial functionality may point to novel breakthroughs (106). To investigate the interplay between irradiation, cancer vaccines, and mitochondria, inactivated vaccines derived from irradiated colon cancer (CT26), lung carcinoma (LL2), and thymoma (EG7) cell lines were shown to inhibit tumor growth in murine models. The study identified oxidation of mtDNA and subsequent activation of the stimulator of interferon genes (STING) pathway as the primary mechanism underlying the observed anti-tumor effects (107). Anti-cancer virotherapy using an oncolytic herpes simplex virus in a 4T1 murine model showed that the treatment increased the expression of pro-tumoral molecules associated with SNS tumoral innervation. When combining the viral therapy with propranolol, a β-blocker, the antitumoral activity was enhanced due to the neuronal suppression and the switch in the infiltrate from perivascular macrophages expressing factors that downregulate T-cell activation, to macrophages with a pro-inflammatory phenotype (108).

Disruption of mitochondrial function through inhibitors of the electron transport chain, as well as through the targeting of specific metabolic pathways involving glucose, fatty acids, and glutamine, has been explored and progressed to clinical trials, yielding promising results (106). Meanwhile, alternative strategies have focused on targeting oncometabolites produced during tumor development, such as succinate, fumarate, and 2-hydroxyglutarate (109).

In efforts to address tumor innervation, bupivacaine, a local anesthetic, was conjugated to polyethylene glycol-based nanoparticles and evaluated in a triple-negative breast cancer (4T1) model to investigate the interplay between neurons and cancer cells. The nanoparticles inhibited the *in vitro* stimulation of new neuronal cells, as well as cancer cell metastasis and survival pathways. In an orthotopic *in vivo* BALB/c mouse model, in which 4T1 tumors were established via subcutaneous instillation into the mammary fat pad, the nanoparticles safely reached the implanted tumors and reduced the infiltration of tumor-associated macrophages, tumoral dimensions, and metastasis while also disrupting neuronal integrity, which was found to be mainly noradrenergic (110, 111). Among the key drivers of axonogenesis, the release of NGF plays a crucial role in promoting the infiltration of neuronal cells into the TME. Targeting NGF and its downstream signaling pathways has been shown to affect tumor size and proliferation in both *in vitro* and *in vivo* models across various cancer types, including prostate, pancreatic, and breast cancers, as well as medulloblastoma, neuroblastoma, and bone sarcoma. Clinical trials targeting NGF have yielded promising results, demonstrating antitumor efficacy with minimal neurological side effects in patients (112, 113). However, reports of adverse effects following anti-NGF-based therapies suggest that further investigation is warranted. Many of these reports have focused on the use of anti-NGF monoclonal antibodies (mAbs) for pain management, primarily in osteoarthritis. Among the anti-NGF mAbs evaluated, bedinvetmab (Librela™, Zoetis) is a canine anti-NGF mAb that inhibits NGF interaction with the tropomyosin receptor kinase A (TrkA) and p75 neurotrophin (p75^NTR^) receptors. Both clinical and laboratory trials demonstrated that bedinvetmab effectively reduced pain while maintaining an acceptable safety profile (114, 115). However, on May 9^th^ of 2025, the use of bedinvetmab was contested in a separate trial, which reported an increase in musculoskeletal adverse events following treatment (116). These findings were subsequently challenged in a commentary published on October 29^th^ of the same year by the Zoetis R&D team (117).

These concerns originated from the failure of tanezumab (Pfizer), another anti-NGF mAb evaluated in clinical trials for the treatment of osteoarthritis. Although significant improvements in pain management and physical function were observed, adverse effects were also reported. Ultimately, the U.S. Food and Drug Administration (FDA) rejected its approval and the drug’s development was discontinued, as the risks of joint destruction and rapidly progressive osteoarthritis were deemed to outweigh the therapeutic benefits (118, 119).

Adrenergic signaling pathways (α- and β-adrenergic receptors) have also been explored as targets for therapies that aim to reduce cancer-neuron interconnection, improving treatment responses and inducing apoptosis pathways, in a tumor-dependent manner (113). Other molecules associated with promoting tumoral innervation have been studied regarding their potential for cancerous proliferation and invasive abilities and could also eventually become targets for novel therapies aimed at modulating TME innervation (112).

Surgical intervention, such as vagotomy, has also been explored as a strategy; however, its effects appear to be highly context-dependent across different cancer types. In a murine model of gastric cancer, vagotomy hindered tumor establishment. Conversely, in models of breast, pancreatic, and liver cancers, the opposite was observed. Similarly, sensory nerve ablation has been investigated in skin and pancreatic cancer models, but findings have likewise been divergent (2, 113).

While therapies targeting mitochondrial function during tumor establishment have been extensively investigated, the inhibition of mechanisms underlying tumor innervation remains relatively underexplored. This raises important questions regarding the feasibility of precision therapies targeting physiological processes such as cellular respiration. Additionally, it remains to be determined whether novel therapies directed at mitochondrial function can be effectively integrated with current advancements in cancer immunotherapy, such as immune checkpoint blockade, at the clinical level. Could therapies targeting mitochondrial metabolism also impede mitochondrial transfer? Given that this transfer is an energy-intensive process, requiring cytoskeletal and membrane remodeling (120), the disruption of key metabolic pathways might interfere with its efficiency. Thus, targeting the metabolic machinery involved in mitochondrial dynamics could potentially hinder intercellular mitochondrial exchange within the tumor microenvironment. Similar questions about the clinical feasibility of viable and non-invasive approaches targeting tumor innervation, alone or in combination with other therapies, are also present. Drugs that target mitochondrial metabolism have been tested concomitantly with other inhibitors, chemotherapeutic drugs, dietetic intervention, and immunotherapeutic approaches (106, 109). Furthermore, could such approaches concurrently inhibit mitochondrial activity within tumor cells or block mitochondrial transfer within the TME? Regardless, the novel findings interconnecting the nervous system and tumor development indicate a potential yet to be explored.

## 6 Tumor-nerve interactions and metabolic reprogramming in aggressive genitourinary cancers

Genitourinary cancers are among the most diagnosed tumors, particularly in men. A burden analysis based on data from the Global Burden of Disease database indicated an overall upward trend in global cases of prostate cancer, which show the highest incidence among genitourinary cancers. Interestingly, this trend appears to be influenced by both age and social factors. In more developed regions, the incidence has either stabilized or declined (121).

**Figure 1:**
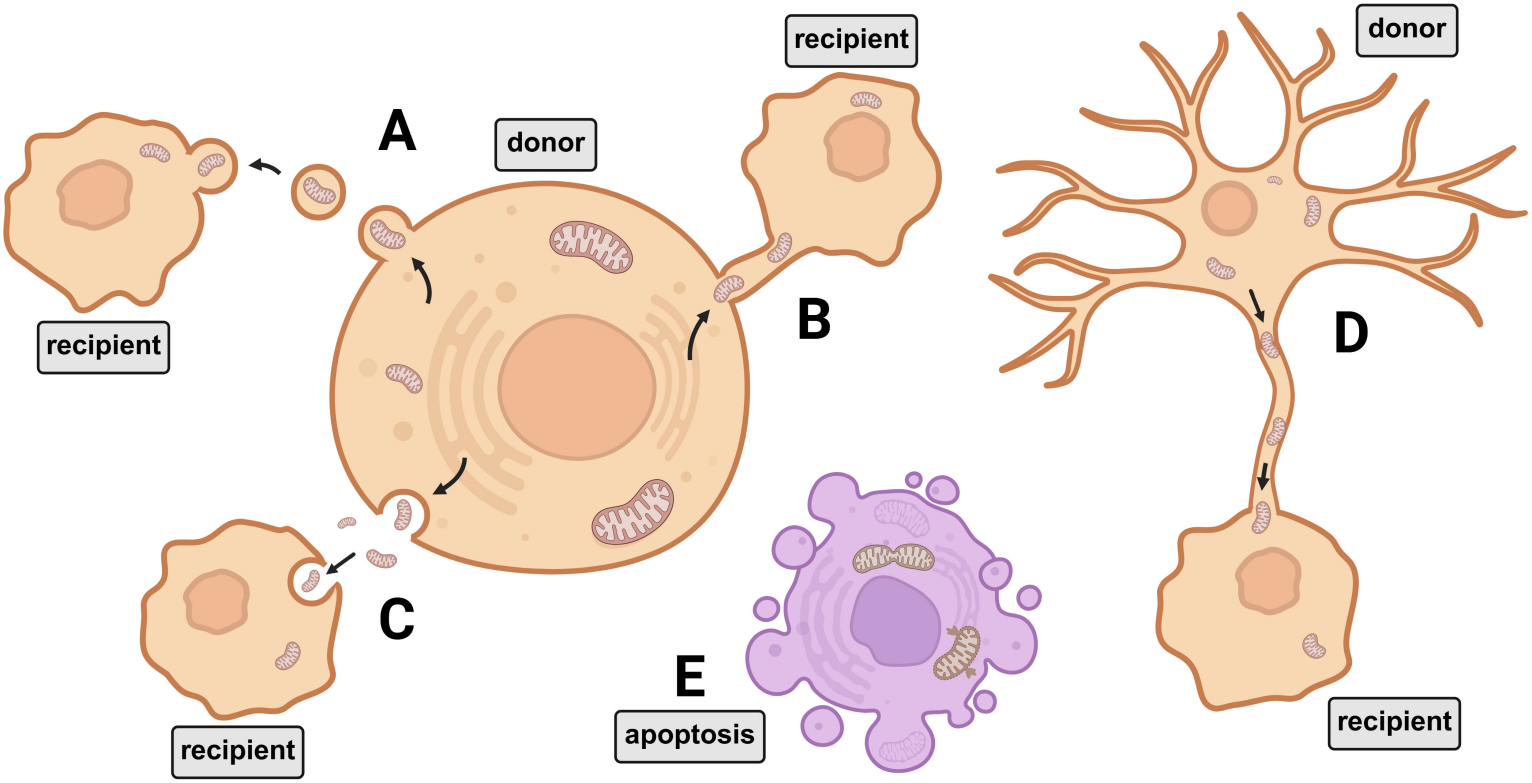
Mitochondrial translocation as a mechanism of metabolic support. Mitochondria can be transferred to cells in need energy supply through mechanisms such as **(A)** microvesicles, **(B)** nanotunnels, **(C)** extrusion and internalization, and **(D)** through dendrites. The activation of these mechanisms promotes a substantial adaptive advantage, increasing the chances of tumor stemness, preventing the release of DAMPs and apoptosis mechanisms **(E)** (4, 93, 94).

Reports have documented mitochondrial transfer and reprogramming as mechanisms contributing to increased invasiveness in bladder cancer. In an *in-vitro* co-culture model using distinct urothelial cancer cell lines (T24 and RT4), spontaneous mitochondrial transfer was observed from the more aggressive T24 cells to the less invasive RT4 cells. As a result, RT4 cells that acquired mitochondria from T24 cells exhibited enhanced invasiveness, accompanied by upregulation of pathways associated with cytoskeleton remodeling. Furthermore, in a subcutaneous xenograft model using athymic BALB/c mice, tumors derived from these modified RT4 cells demonstrated increased size compared to those formed by the original RT4 cell line (122). A correlation between mitochondrial transfer and tumor aggressiveness has also been observed in prostate cancer models. Both *in-vitro* and *in-vivo* xenograft assays using immunodeficient (SCID-bg/bg) mice demonstrated that mitochondria can be transferred from cancer-associated fibroblasts to prostate cancer cells. This mitochondrial transfer was shown to induce metabolic reprogramming, which in turn promoted increased invasion and malignant potential of the cancer cells (123). Another pro-oncogenic feature in urothelial cancer associated with mitochondria involves the metabolic reprogramming of platelets. The role of platelets in cancer progression is well established, and thrombocytosis, which is partly due to increased activation signals derived from tumor cells, has been linked to poorer clinical outcomes. In platelets isolated from urothelial cancer patients, mitochondrial analysis revealed elevated oxidative stress and a shift in metabolic preference favoring the glycolytic pathway (124). **Figure 1** illustrates the main mechanisms by which mitochondria are transferred between cells to support energy homeostasis and promote tumor stemness.

Therapies targeting nerve development have also been investigated in models of genitourinary cancers. Perineural invasion is a hallmark of aggressive prostate cancer, providing both survival advantages and pathways for metastasis. *In vitro* and histological studies have demonstrated that prostate cancer can induce neurogenesis and axonogenesis through the action of S4F, suggesting that PSNS innervation is closely associated with tumor aggressiveness and the hormonal regulation of prostate cancer (10, 125). Pro-nerve growth factor (proNGF), a molecule associated with axonogenesis, was found to be overexpressed and secreted by tumor cells in prostate cancer. Immunohistochemistry assays further demonstrated that elevated proNGF levels correlated with Gleason scores ≥ 8 (126). Another neurotropic molecule, granulocyte colony-stimulating factor (G-CSF), was shown to promote autonomic innervation in an orthotopic mouse model of prostate cancer, which was associated with increased tumor invasion and spread (127). Collectively, the results indicate that a range of molecular factors may contribute to tumor-associated nerve infiltration and worse prognosis in clinical settings.

Additionally, the SNS may contribute to prostate cancer progression. In murine models, noradrenaline-induced adrenergic signaling in endothelial stromal cells enhanced vascularization of the tumor microenvironment, which was associated with increased tumor development and invasion. Notably, mitochondrial reprogramming accompanied this angiogenic process, with a shift from normal glycolysis towards oxidative phosphorylation. Inhibition of this metabolic switch suppressed tumor vascularization, angiogenesis, perineural invasion, and disease progression. These findings suggest that mitochondrial reprogramming of endothelial stromal cells, driven by adrenergic signaling, may represent a key mechanism underlying prostate cancer aggressiveness (128). When using the transgenic Hi-Myc mice model to establish prostatic intraepithelial neoplasia, either pharmacological inhibition of the SNS or surgical ablation of the hypogastric nerves suppressed tumor formation when performed at early disease stages. At later stages, PSNS innervation emerged as the primary driver of metastasis; in these cases, interference with cholinergic receptors led to improved survival outcomes. These findings were supported by histological analyses of clinical samples, which demonstrated that increased innervation of the TME and its associated stroma correlated with poorer prognoses (129).

Another denervation study using a rat model demonstrated the therapeutic potential of nerve inhibition in prostate cancer. Both surgical and chemical denervation, the latter achieved through administration of botulinum toxin type A, resulted in tumor reduction in an orthotopic prostate cancer model using male NIH-Foxn1^rnu^ nude rats. Furthermore, transcriptional analysis revealed downregulation of translational machinery and metabolic pathways in both tumor and stromal compartments following denervation (130). As it relates to the clinical aspect, given that β-blockers inhibit adrenergic activity through binding in the β2-adrenergic receptor, Norwegian cohort studies involving patients with prostate cancer undergoing androgen-deprivation therapy found that β-blocker use was associated with reduced mortality among patients classified as high-risk, or those with existing metastases (131, 132).

Denervation models of bladder cancer using female B6 mice demonstrated that the presence of nerve fibers suppressed the immune response within the TME, leading to increased tumor growth, primarily through the overexpression of the PD-1 immune checkpoint. The same study also analyzed histological samples and transcriptomic data from bladder cancer patients obtained from public databases. It revealed that individuals with a higher density of neural terminations exhibited a reduced population of immune cells within the TME. Furthermore, patients with elevated expression of post-synaptic proteins showed decreased overall survival rates; however, they also demonstrated improved responses to PD-1–targeted therapies. The long-term use of metoprolol also correlated with suppression of tumor growth (133). The interconnection between immune checkpoint expression and tumoral innervation broadens the potential for novel therapeutic strategies targeting both processes. A study from our group (134) reports distinct expression profiles of immune suppression markers, including PD-1, CD163, and FOXP3, between primary tumoral and metastatic cells in prostate adenocarcinoma. These findings support the hypothesis that, once metastatic cells exit the immunosuppressive milieu of the primary tumor microenvironment, they become more vulnerable to Immunogenic Cell Death (ICD), revealing a therapeutic window for targeted interventions. Previous approaches targeting tumor innervation have already been tested in association with other therapies such as PD-1 and CTL-4 inhibitors, radiotherapy, NGF inhibitors, anesthetics, chemotherapeutic drugs, and COX-2 inhibitors (5, 135, 136).

Overall, in genitourinary cancers, the survival advantages conferred by mitochondrial acquisition and the increased aggressiveness observed in innervated tumors suggest novel avenues for therapeutic intervention. These findings highlight the potential of targeting both mitochondrial dynamics and tumor innervation as part of clinical strategies. However, current innervation-focused studies have predominantly concentrated on prostate and bladder cancer, raising important questions about whether similar mechanisms may be involved in other common genitourinary malignancies, such as renal cancer. Notably, large-scale cohort studies have indicated that the use of β-blockers may be associated with improved prognosis, supporting the potential utility of innervation-directed therapies to mitigate the pro-tumorigenic effects of neural signaling on progression and metastasis. Furthermore, it remains to be clarified whether nerve-associated invasion mechanisms are mechanistically linked to metabolic reprogramming or represent distinct, parallel features of tumor aggressiveness. For example, perineural invasion is commonly observed in certain tumors, such as prostate cancer. This raises the question of whether it is driven by the neurotrophic behavior of prostate cancer cells or by the anatomical characteristics of the prostate, including its rich neural innervation. As more pathways are uncovered, challenges in establishing and evaluating those models of complex interactions arise. Nevertheless, these emerging insights offer promising opportunities for the development of more effective, targeted, and combinatorial treatment strategies to enhance clinical outcomes in genitourinary cancers.

## 7 Future perspectives

With emerging evidence over the past few years, the intersection of neuroscience, oncology, and immunology has become deeper, broadening both conceptual frameworks and therapeutic possibilities. However, despite this promising and exciting integration, several challenges remain in implementing a truly multidisciplinary approach. Advancing our understanding of tumor innervation will require the development and refinement of robust experimental models.

Nevertheless, the neuronal cross-talk, particularly in light of recent findings on mitochondrial transfer from nerves and its role in metabolic reprogramming and increased tumor aggressiveness, opens the door for the possibilities of the development of novel therapeutic strategies that target these interconnected processes concurrently, rather than addressing them as isolated features (4). As a result, a few questions arise, mainly: what are the full implications provided by the metabolic reprogramming induced by tumor innervation, and how could we address the presented issues? Although further research is necessary to fully elucidate these mechanisms, by combining the evidence that points towards the immunological modulation of the TME, associated with the metabolic advantages for proliferation and dissemination, the role of tumor innervation may be more significant than previously recognized, warranting greater attention in both basic research and therapeutic development. β-blockers are adrenergic inhibitors and have been investigated in different clinical settings, yielding mostly promising results (137–139). The complexity observed between the effects of innervation of different types of tumors may point to a pharmaceutical approach rather than a surgical one. Since vagotomy and nerve ablation have shown conflicting results regarding outcomes following the procedure (2, 113), more evidence is required to develop viable clinical options to tackle the tumoral innervation.

Despite promising progress, therapies targeting mitochondrial metabolic pathways continue to face significant bottlenecks. One major challenge is specificity, as many metabolic enzymes have functional isoforms, in addition to the difficulty in inducing the necessary structural alterations for therapeutic efficacy. Additionally, cancer cells often compensate by activating alternative metabolic routes, thereby reducing the overall effectiveness of these treatments. Systemic toxicity also remains a concern for several mitochondrial-targeting compounds. Nevertheless, restoring mitochondrial function may resensitize tumor cells to apoptosis and activate ICD pathways, contributing to their elimination. Another therapeutic strategy involves disrupting key metabolic pathways to slow cancer progression; however, this approach also raises systemic toxicity concerns for patients, underscoring the need for more selective and tolerable interventions (106, 140). Although potential therapies aimed at correcting genomic alterations in mtDNA remain largely unavailable, continued advancements in genetic engineering may eventually render such approaches feasible (109).

**Figure 2:**
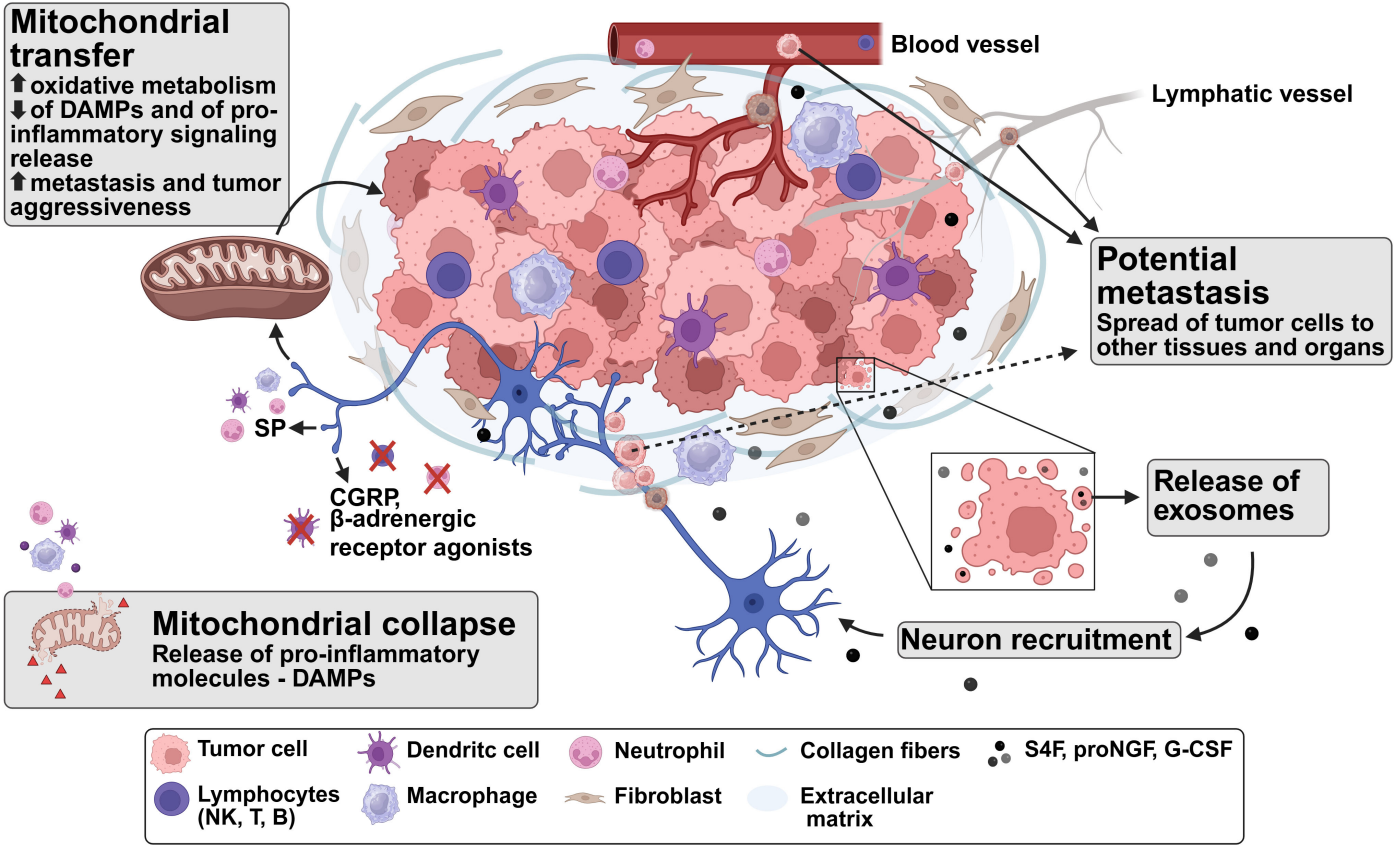
The mitochondria–nerve–immunity triad in tumor microenvironment.

A summary of the findings discussed in this review is presented in **Figure 2**, which synthesizes the correlations between tumoral innervation and mitochondrial transfer in the context of survival advantages and tumor aggressiveness. Overall, the process of tumor innervation, which was once largely overlooked, has now gained significant attention in recent years, with emerging evidence highlighting its critical role in cancer behavior and resistance, underscoring the need for a deeper understanding of the underlying mechanisms, including potential correlations between the directionality of the neurogenesis with the peritumoral innervation patterns and the lipidic composition of nerves. These questions prompt a broader reconsideration of the role of innervation during cancer development and progression. Further studies are essential to elucidate the contribution of the nervous system to cancer development and progression, which may ultimately uncover novel therapeutic targets.

## 8 Conclusions

An intriguing feature of genitourinary cancers is the naturally innervated physiological environment of these sites, particularly in prostate cancer, where perineural invasion might represent a hallmark of aggressive tumors. Both *in vitro* and *in vivo* models of prostate and bladder cancer, mitochondrial transfer to tumor cells and TME innervation have been associated with enhanced tumor aggressiveness, particularly in relation to their roles in immunomodulation, invasiveness, and resistance to existing treatments. Considering the recently uncovered link between innervation and metabolic reprogramming, alongside its pro-oncogenic potential, there is a compelling rationale for the development of novel studies and translational therapies targeting both TME innervation and mitochondrial transfer–mediated metabolic reprogramming. Such strategies, whether deployed as monotherapies or in combination with existing approaches such as immune checkpoint blockade, hold promise for more effective treatment strategies and potentially improved clinical outcomes in genitourinary cancers.
